# Molecular typing of rotaviruses in gastroenteritis cases detected in foreign-born children aged 0–5 years

**DOI:** 10.1128/spectrum.02406-25

**Published:** 2026-05-05

**Authors:** Salam Charbak, Mustafa Sağlam, Tekin Karsligil

**Affiliations:** 1 Department of Medical Microbiology, Institute of Health Sciences, Gaziantep University, Gaziantep, Turkey; 2 Faculty of Medicine, Department of Medical Microbiology, Gaziantep University, Gaziantep, Turkey; 3 Faculty of Medicine, Department of Medical Microbiology, University of Kyrenia, Kyrenia, Cyprus; Purdue University, West Lafayette, Indiana, USA

**Keywords:** gastroenteritis, rotavirus, genotyping, PCR, Syrian

## Abstract

Rotavirus is one of the causes of viral gastroenteritis in childhood. It is one of the most common causes of mortality and morbidity in developing countries. The objective of this study was to determine the molecular typing of rotaviruses in gastroenteritis cases detected in foreign-born children aged 0–5 years. Out of the 120 stool samples received by our laboratory between May 2022 and November 2023, samples from 74 patients who tested positive for rotavirus were included in the study. The study was carried out in the Microbiology Laboratory of Gaziantep University Şahinbey Research and Application Hospital. One Step RT-PCR was used for genotyping. Positivity rates were 32.4% for P4, 17.6% for P6, and 37.8% for P8. G genotypes were G1 in 32.4%, G4 in 18.9%, and G9 in 23% of the samples. P9 genotype was found in 9% of girls and 22% of boys (*P* < 0.05). G1P[8] and G2P[4] genotypes were the most common genotypes in cases with rotavirus infection. Although there were common serotypes in the region, different serotypes were found in foreign-born children.

**IMPORTANCE:**

Rotavirus gastroenteritis continues to pose a significant health challenge in early childhood, especially in developing regions where the burden of illness remains high. Understanding which rotavirus genotypes are circulating in a community is important for tracking epidemiological trends and improving preventive strategies, including vaccination programs. In areas such as Gaziantep, where migration has led to a growing population of foreign-born children, information on the molecular characteristics of rotavirus strains is still limited. By examining genotype distribution among foreign-born children aged 0–5 years, this study provides valuable data on circulating strains and helps clarify regional patterns, contributing to more effective surveillance and better-informed public health measures.

## INTRODUCTION

Gastroenteritis is caused by many species in different pathogen groups, including bacteria, parasites, and viruses. Among viral agents, rotavirus (RV) is one of the most common pathogens responsible for acute gastroenteritis cases (1). Rotaviruses, which are primarily transmitted via the fecal-oral route, affect the majority of children worldwide before the age of 3 years and newborns in most developing countries (2). Rotavirus infection remains a major global health concern for young children. Each year, millions of children under the age of 5 become infected with rotavirus, and despite improvements in prevention and treatment, the infection still leads to an estimated 122,000–215,000 deaths worldwide in this age group. These numbers highlight the continuing burden of rotavirus, particularly in settings where access to vaccination and adequate medical care is limited (3). Although viral strains vary significantly, five serotypes are responsible for the majority of human rotavirus disease. The serotype of rotavirus is determined by an internal capsid protein called VP6. This protein is found inside the virus and plays a role in the recognition of the virus by the immune system. The serotypes of rotavirus are usually referred to as P (I, II, III, IV, V, VI, VII, VIII, IX, and X). These serotypes refer to genetic differences in the G (glycoprotein) and P (protein) proteins on the surface of the virus, which are targeted by antibodies. The virus consists of three protein shells surrounding 11 segments of double-stranded RNA: the outer capsid, inner capsid, and inner core. The outer capsid proteins VP4 and VP7 are neutralization antigens and define the P and G serotypes, respectively. The inner capsid structural protein VP6 is a subgroup antigen (4).

RV, the subject of this study, is a member of the Reoviridae family. RV consists of an 11-segmented genome of double-stranded RNA and a three-layered protein structure surrounding this genome. The viral genome encoding 12 proteins, 6 structural and 6 non-structural, is surrounded by a sheath formed by the structural proteins VP2 in the inner layer, VP6 in the middle layer, and VP7 and VP4 in the outer layer. VP6 protein is the antigen specific to groups and subgroups and is divided into eight groups from A to H on the basis of differences in this antigen (5). While group A, B, and C RVs cause infection in humans, group A RVs stand out as the most common group in childhood diarrhea. VP4 and VP7 proteins forming the viral capsid are antigens that trigger neutralizing antibody production and determine RV P and G serotypes and genotypes, respectively (6). So far, 42 G and 58 P genotypes have been reported (7).

During the replication of a large number of RVs that settle in the small intestine of the host in intestinal cells, point mutation, reassortment, and new genetic rearrangements can develop due to their segmental genomes, and as a result, many genotype variations of RV can occur (8). Gene rearrangement is mostly seen in the 11^th^ segment encoding the non-structural NSP5 and NSP6 proteins of the virus, while it is seen in the 5^th^ to 10^th^ segments at lower rates (9). Another factor that is effective in the emergence of new phenotypes is mixed infections (10).

Currently, four oral rotavirus vaccines (RotaTeq [Merck & Co., USA], Rotarix [GlaxoSmithKline, Belgium], Rotavac [Bharat Biotech, India], and Rotasiil [Serum Institute of India, India]) have received prequalification from the World Health Organization for global use. Pentavalent RotaTeq reassortant (containing genetic material derived from two or more similar viruses) vaccine contains G1, G2, G3, G4, and P1[8] viral surface antigens. Monovalent Rotarix vaccine produced especially for G1 type and containing G1P1AP[8] antigen is effective in most of the G types (11, 12).

In conclusion, RVs are a global health problem causing great economic and life losses all over the world. The presence of many types due to its genome characteristics and the continuous emergence of new genotypes increase the importance of vaccines developed and to be developed against this virus.

## MATERIALS AND METHODS

### Research samples

Stool samples of 120 foreign-born children aged 0–5 years with gastroenteritis who were sent to the microbiology laboratory of Migrant Health Center and Gaziantep University Şahinbey Research and Application Hospital between May 2022 and November 2023 were included in our study.

### Immunochromatographic method

In our study, 120 stool samples were investigated for the presence of rotavirus antigen by immunochromatographic method using Laboquick Rotavirus Ag Test Kit (Köroğlu, Turkey) with 98.4% sensitivity and 98.9% specificity, and stored at −20°C until genotyping test was performed.

### Molecular method

#### Nucleic acid extraction from fecal samples

Nucleic acid extraction from fecal samples was performed using QIAamp Viral RNA Mini Kit (Qiagen, Germany) according to the manufacturer’s instructions. Seventy-four patient samples that tested positive for rotavirus using the immunochromatographic method were included in the nucleic acid isolation procedure.

#### Complementary DNA synthesis

Complementary DNA (cDNA) was obtained from the RNA isolation products using miRCURY LNA RT Kit (Qiagen, Maryland, USA). For cDNA, 2 µL of 5× miRCURY RT reaction buffer, 5 µL of RNase-free water, 1 µL of 10× miRCURY RT enzyme mix, and 2 µL of template RNA (5 ng/µL) were converted into cDNA using a PCR protocol of 60 min at 42°C (reverse transcription step), 5 min at 95°C (inactivation of reaction), and indefinite storage at 4°C (storage).

#### Genotyping

Forward and reverse primers of VP4 and VP7 genes (Macrogen, Republic of Korea) were designed to identify rotavirus G and P types (Table 1) (13). For typing, miRCURY LNA SYBR Green PCR Kit (Qiagen, Maryland, USA) was used. Five microliters of 2× miRCURY SYBR Green Master Mix, 2 µL of forward primer, 2 µL of reverse primer, 6.5 µL of DNase/RNase-free H_2_O, and 4.5 µL of cDNA were used for typing on Rotor Gene Q 5 Plex Real-Time PCR (Qiagen, Hilden, Germany) using the PCR protocols given in Table 2.

**TABLE 1:** G and P consensus and type-specific primers (13)

Primers	Sequences (5′–3′)	Amplicon sizes
VP4-F	TATGCTCCAGTNAATTGG	663
VP4-R	ATTGCATTTCTTTCCATTAATG	
VP7-F	ATGTATGGTATTGAATATACCAC	881
VP7-R	AACTTGCCACCATTTTTTCC	
G1	CAAGTACTCAAATCAATGATGG	618
G2	CAATGATATTAACACATTTTCTHT	521
G3	ACGAACTCAACACGAGAGG	682
G4	CGTTTCTGGTGAGGAGTTG	452
G8	GTCACACCATTTGTAAATTCG	754
G9	CTTGATGTGACTAYAAATAC	179
G10	ATGTCAGACTACARATACTGG	266
P[4]	CTATTGTTAGAGGTTAGAGTC	362
P[6]	TGTTGATTAGTTGGATTCAA	146
P[8]	TCTACTGGRTTRACBTGC	224
P[9]	TGAGACTGCAATTGGAC	270
P[10]	ATCATAGTTAGTAGTCGG	462
P[11]	GTAAACATCCAGAATGTG	191

**TABLE 2:** Real-time PCR protocol

Gene regions	Hot start activation	Denaturation	Annealing and extension
VP4, VP7-G9	95°C / 2 min	95°C / 10 s	56°C / 60 s
		45 Cycles	
VP7, VP4-P8, VP4-P9, VP7-G2, VP7-G8, VP7-G10	95°C/2 min	95°C/10 s	53°C/60 s
		45 cycles	
VP4-P4, VP4-P6, VP4-P10	95°C/2 min	95°C/10 s	52°C/60 s
		45 cycles	
VP4-P11	95°C/2 min	95°C/10 s	50°C/60 s
		45 cycles	
VP7-G1	95°C/2 min	95°C/10 s	55°C/60 s
		45 cycles	
VP7-G3, VP7-G4	95°C/2 min	95°C/10 s	58°C/60 s
		45 cycles	

Regions were identified using G primers (G1, G2, G3, G4, G8, G9, and G10) with the VP7-R consensus primer and P primers (P4, P6, P8, P9, P10, and P11) with the VP4-F consensus primer.

### Statistical analyses

The data obtained from the study were analyzed using SPSS 18.0 software. Percentage values were given as descriptive statistics. Chi-square analysis was used to determine the relationship between categorical variables, and *P* < 0.05 was accepted as significant.

## RESULTS

A total of 120 foreign-born children were evaluated in the study. The rotavirus antigen test was found to be positive in 74 (61.7%) patients. Of the children included in the study, 64.9% (*n* = 48) were under 2 years of age, 35.1% (*n* = 26) were between 2 and 5 years of age; 55.4% (*n* = 41) were boys, and 44.6% (*n* = 33) were girls.

**Fig 1:**
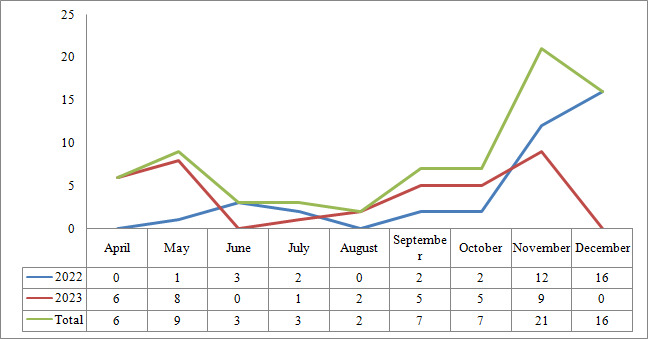
Rotavirus positivity rates by years and months.

Samples were included in the study between 2022 and 2023. A total of 38 patients were positive in 2022, and 36 patients were positive in 2023. In 2022, the highest positivity rate was in December with a rate of 42.1%, while in 2023, the highest positivity rate was in November with a rate of 25%. The highest rate of positivity was detected in November (28.4%) and December (21.6%), while the lowest rates were detected in June–July (4.1%) and August (2.7%). No samples were collected in February and March due to the February 6 earthquake (Fig. 1).

As a result of our study, VP4 genotype was detected in 91.9% (*n* = 68) of foreign-born children and not detected in 6 children, while VP7 genotype was detected in 79.7% (*n* = 59) of children and not detected in 15 children. The most common VP4-P genotype detected in foreign-born children was P8 (37.8%), and the most common VP7-G genotype was G1 (32.4%) (Table 3).

**TABLE 3:** Comparison of VP4-P and VP7-G genotypes according to gender

		Girl, *n* (%)	Boy, *n* (%)	Total
VP4-P	P4	15 (45.4)	9 (22.0)	24 (32.4)
	P6	4 (12.2)	9 (21.9)	13 (17.6)
	P8	12 (36.3)	16 (39.0)	28 (37.8)
	P9	3 (9.0)	9 (22.0)	12 (16.2)
	P10	3 (9.0)	0 (0.0)	3 (4.1)
	P11	0 (0.0)	3 (7.3)	3 (4.1)
VP7-G	G1	12 (36.3)	12 (29.2)	24 (32.4)
	G2	4 (12.1)	3 (7.3)	7 (9.5)
	G3	1 (3.0)	0 (0.0)	1 (1.4)
	G4	6 (18.2)	8 (19.5)	14 (18.9)
	G8	0 (0.0)	0 (0.0)	0 (0.0)
	G9	6 (18.2)	11 (26.8)	17 (23.0)
	G10	3 (9.1)	3 (7.3)	6 (8.1)

When P genotypes were evaluated according to gender, P9 genotype was found in 9% of girls and 22% of boys. In addition, 36.3% of girls and 22% of boys were P8 and P4 positive, respectively. G1 was positive in 36.3% of girls and 29.2% of boys, G4 in 18.2% of girls and 19.5% of boys, G9 in 18.2% of girls and 26.8% of boys (Table 3).

**Fig 2:**
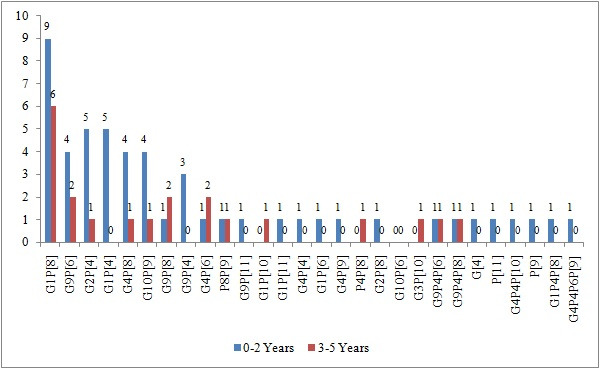
Distribution of rotavirus G and P genotype combinations according to age groups.

No significant difference was found between the distribution of rotavirus G and P genotype combinations according to age groups. However, no G and P genotype combination was detected in 83.3% of foreign-born children under 2 years of age and 92.3% of those aged 2–5 years. In contrast, a combination of G1P[8] genotypes was detected in nine children (14.3%) under 2 years of age (Fig. 2).

When examining the distribution of rotavirus mixed multiple genotypes identified in this study, the G1P[8] genotype was the most frequently detected type in 15 patients (20.3%; 9 boys, 6 girls). This was followed by the G9P[6] genotype in 6 patients (5 boys, 1 girl) and the G2P[4] genotype in 6 patients (8.1%; 3 boys, 3 girls). Additionally, the G1P[4], G4P[8], and G10P[9] genotypes were each detected in 5 patients (6.8%), with the G1P[4] genotype identified in 1 boy and 4 girls, the G4P[8] genotype in 3 boys and 2 girls, and the G10P[9] genotype in 2 boys and 3 girls. Among the genotypes with lower frequency, the G9P[4]P[6] and G9P[4]P[8] genotypes were detected in 2 patients each (2.7%; both male). The G4P[4]P[6]P[9] genotype was detected in 1 patient (1.4%; girl) (Table 4).

**TABLE 4:** Comparison of the distribution of rotavirus G, P, and mixed multiple genotype combinations according to gender

Genotype combinations	Boy, *n* (%)	Girl, *n* (%)	Total, *n* (%)
G[4]	1 (1.4)	1 (1.4)	2 (2.7)
P[9]	0 (0.0)	1 (1.4)	1 (1.4)
P[11]	0 (0.0)	1 (1.4)	1 (1.4)
G1P[8]	9 (12.2)	6 (8.1)	15 (20.3)
G9P[6]	5 (6.8)	1 (1.4)	6 (8.1)
G2P[4]	3 (4.1)	3 (4.1)	6 (8.1)
G1P[4]	1 (1.4)	4 (5.4)	5 (6.8)
G4P[8]	3 (4.1)	2 (2.7)	5 (6.8)
G10P[9]	2 (2.7)	3 (4.1)	5 (6.8)
G9P[8]	2 (2.7)	1 (1.4)	3 (4.1)
G9P[4]	2 (2.7)	1 (1.4)	3 (4.1)
G4P[6]	2 (2.7)	1 (1.4)	3 (4.1)
G9P[11]	1 (1.4)	0 (0.0)	1 (1.4)
G1P[10]	0 (0.0)	1 (1.4)	1 (1.4)
G1P[11]	1 (1.4)	0 (0.0)	1 (1.4)
G1P[6]	0 (0.0)	1 (1.4)	1 (1.4)
G4P[9]	1 (1.4)	0 (0.0)	1 (1.4)
G2P[8]	0 (0.0)	1 (1.4)	1 (1.4)
G10P[6]	1 (1.4)	0 (0.0)	1 (1.4)
G3P[10]	0 (0.0)	1 (1.4)	1 (1.4)
G9P4P[6]	2 (2.7)	0 (0.0)	2 (2.7)
G9P4P[8]	2 (2.7)	0 (0.0)	2 (2.7)
G4P4P[10]	1 (1.4)	0 (0.0)	1 (1.4)
G1P4P[8]	0 (0.0)	1 (1.4)	1 (1.4)
G4P4P6P[9]	0 (0.0)	1 (1.4)	1 (1.4)
P8P[9]	2 (2.7)	0 (0.0)	2 (2.7)
P4P[8]	0 (0.0)	1 (1.4)	1 (1.4)
P4G[4]	0 (0.0)	1 (1.4)	1 (1.4)
Total	41 (55.4)	33 (44.6)	74 (100.0)

## DISCUSSION

Rotaviruses are one of the main causative agents of severe gastroenteritis in infants and young children on a global scale (13). More than one-third of diarrhea-related deaths and approximately 5% of deaths in children under 5 years of age are associated with rotavirus infections (13). In both developed and developing countries, these viruses are responsible for 25%–50% of hospitalizations due to diarrhea (14). While the mortality rate due to rotavirus infection is low in regions where health services are developed, it has been reported that the mortality rate increases up to 85% in South Asia and Sub-Saharan Africa (15).

Rotaviruses are divided into seven main groups (A–G) according to the antigenic and genetic properties of the inner capsid protein VP6 (16). The structural proteins VP4 and protease-sensitive VP7 in the outer capsid define G and P genotypes, respectively (7). The host specificity of VP4 and VP7 proteins is critical in terms of virulence level and neutralizing antibody response (7, 17).

Due to the segmental structure of the viral genome, reassortment events occur in which P and G genotypes are produced between two strains infecting the same host (17). So far, 42 G and 58 P genotypes have been reported by the RVA Classification Working Group (18). The prevalence of genotypes varies depending on years and geographical regions, and only a few G-P combinations, especially G1P[8], G2P[4], G3P[8], G4P[8], and G9P[8], are reported to be common in humans worldwide (19, 20). On the other hand, rarer genotypes such as G12P[8], G12P[6], G2P[8], G4P[6], and G3P[6] have been reported at lower rates in different countries (21–23). There are no studies examining RV genetic diversity in foreign-born children in Gaziantep and the surrounding areas. There is also no similar study conducted in Syria. This region of Turkey is a particularly densely populated area of foreign-born asylum-seekers, who often live in crowded and communal settings, and their interactions are mostly among themselves. Some of these individuals frequently travel to Syria and are in constant contact with this country. Therefore, in our study, we investigated the possibility of this group being infected with a different serotype/genotype due to their genetic and structural characteristics.

Our study aims to provide important information on the antigenic distribution of rotavirus vaccines by revealing the genotype distribution in foreign-born children. The distribution of different combinations of genotypes was also determined.

In this study, the genotype distribution of 74 foreign-born children aged 0–5 years who were found to be rotavirus positive was investigated. While 64.9% of the children included in the study were under 2 years of age, 35.1% were between 2 and 5 years of age. Rotavirus is the most common and leading cause of childhood diarrhea worldwide (24), and almost all children are infected with rotavirus by the age of 5. Studies have reported that approximately one-third of infections occur in children under 2 years of age (25–27). By the age of 5, it is estimated that 1 in 5 children will present to an outpatient health care facility, and 1 in 60 children will be hospitalized due to rotavirus infection (28). Children under 2 years of age are at risk of being infected with rotavirus, which is an extremely resistant virus in environmental conditions, because they have just started walking and interact more with their environment. In this respect, the results obtained in our study are considered as expected findings.

Of the foreign-born children included in the study, 41 (55.4%) were boys, and 33 (44.6%) were girls. In previous studies, it was reported that rotavirus positivity rates were similar in boys and girls (25–27). The highest positivity rates were observed in November (28.4%) and December (21.6%), and the lowest rate was observed in August (2.7%). Due to the February 6 earthquake disaster, no samples could be collected in February and March. In Turkey, it has been reported that rotavirus infections are most common between the beginning of September and the end of May, and the highest infection rates occur in the first 4 months of the year (January, February, March, and April) (25, 29, 30). In a study conducted across Turkey by Durmaz et al. (25), it was reported that the highest positivity rate was in March with 17.1%, followed by January and February with 14.4% and 13.1%, respectively. Kahraman et al. (31) reported that 35.9% of rotavirus positive cases were seen in spring, 24.6% in fall, 19.7% in winter, and 19.7% in summer.

In our study, VP4 genotype was detected in 91.9% of foreign-born children, and similarly, the proportion of patients with VP7 genotype was also high (79.7%). P[8] genotype was detected in 37.8%, P[4] in 32.4%, and P[6] in 10.8% of the foreign-born children included in the study. In a study conducted by Durmaz et al. (25) in Turkey, it was reported that the predominant P genotype was P[8] (79.4%), followed by P[4] (19.5%), P[9] (0.43%), P[6] (0.3%), P[11] (0.12%), and P[10] (0.06%) genotypes, respectively. In our study, it was observed that the predominant genotype among foreign-born children was P[8], but the rate was lower, whereas P[6] genotype was detected at a higher rate.

In our study, G9 positivity was detected in 23% and G4 positivity in 18.9% of foreign-born children, and G8 positivity was not detected in any of the patients. In the study by Kahraman et al. (31), G type genotype was determined in 80 of 81 patients, and seven different G types (G1, G2, G3, G4, G4, G9, G10, and G12) were reported. In this study, G1 was found in 37%, G9 in 9.9%, and G4 in 6.2%. Although this order is compatible with our findings, the rates differ. In the study conducted by Durmaz et al. (25), both G and P genotypes were identified in a total of 1,638 samples. In this study, G9 genotype was detected in 48.7%, followed by G1 in 25.9%, G2 in 16.2%, G3 in 4.3%, G4 in 2.7%, and G8 in 0.9% of the patients. While G9 genotype was found to be dominant in the study, the dominant genotype in our study was determined as G1.

When evaluating the distribution of rotavirus mixed genotypes identified in this study, it is noteworthy that the G1P[8] genotype was the most frequently identified type (20.3%). This finding is consistent with G1P[8] being reported as the dominant genotype in many geographic regions, indicating that this genotype continues to be epidemiologically important among circulating rotavirus strains (32). The G1P[8] genotype is followed by the G9P[6] and G2P[4] genotypes at lower rates, suggesting that rotavirus genotype distribution exhibits a heterogeneous structure and that multiple genotypes circulate simultaneously in the region. However, the detection of G1P[4], G4P[8], and G10P[9] genotypes at similar rates (6.8%) highlights the need to consider the presence of rare or emerging genotypes. Multiple genotype combinations such as G9P[4]P[6], G9P[4]P[8], and G4P[4]P[6]P[9], which were identified at lower frequencies, indicate that reassortment mechanisms may be active in rotaviruses. This situation may lead to increased viral diversity and potentially increase the importance of vaccine efficacy and epidemiological surveillance studies. In the study by Durmaz et al. (25), the mixed infection rate in Turkey ranged between 2.4% and 26%. Our results are in parallel with the rates of uncommon and mixed infections ranging between 3.7% and 15% in other countries. A systematic review synthesizing global, longitudinal data from 2006 to 2010 by Dóró et al. (33) found no consistent genotype prevalence pattern indicating selection pressure associated with vaccine use during this time period. The review reported that human rotavirus infections worldwide were largely composed of six major genotypic combinations: G1P[8], G2P[4], G3P[8], G4P[8], G9P[8], and G12P[8]. These findings suggest that rotavirus genotype distribution was relatively stable in the early period and that vaccination programs did not have a significant guiding effect on genotype composition. The findings obtained in this study largely coincide with the results of the aforementioned systematic review.

In conclusion, the findings of this study, consistent with global data from the early post-licensing period, show that the G1P[8] genotype remains dominant, but also that genotype diversity and reassortment potential continue. This situation highlights the need for continuous and detailed genotypic surveillance in evaluating the effectiveness of rotavirus vaccination programs and in the early detection of future genotypic changes.

In our study, when P genotypes were compared with gender, the P9 genotype was detected in 9% of foreign-born girls and 22% of foreign-born boys, and this difference was found to be significant. In addition, 36.3% of girls and 22% of boys were positive for P4 and P8 genotypes, respectively. When G genotypes were compared with gender, no significant difference was found between foreign-born girls and boys in terms of G genotypes. While 36.3% of girls had G1, 18.2% G4, 18.2% G9, 9.1% G10, 12.1% G2, and 3% G3 genotypes, 29.2% of boys had G1, 19.5% G4, 26.8% G9, 7.3% G10, and 3% G2 genotypes. Durmaz et al. (25) reported that G9P[8] and G1P[8] genotypes were the most common and second most common genotypes in all age groups, and genotypes were detected with similar frequency in both genders. These results, in parallel with our study, show a similar trend in the distribution of genotypes between the genders.

In our study, rotavirus G and P genotype combinations were detected in 87.9% of girls and 85.4% of boys. In girls, G1P[8], G1P[4], and G2P[4] combinations were observed in 9.5%, 6.3%, and 4.8%, respectively. In boys, G1P[8], G1P[4], G2P[4], and G9P[6] combinations were observed in 14.3%, 1.6%, 4.8%, and 7.9%, respectively. No significant difference was found between boys and girls in terms of G and P genotype combinations. In the study conducted by Durmaz et al. (25), the rates of G9P[8], G1P[8], G2P[8], and G2P[4] combinations in boys were found to be 39%, 22%, 8%, and 6%, respectively, while these rates in girls were 43%, 21%, 11%, and 7%, respectively. In this study, it was reported that there was no significant difference between boys and girls in terms of combinations.

When the distribution of VP4-P and VP7-G genotypes was compared according to months, no significant difference was observed. In the study conducted by Durmaz et al. (25), no significant difference was found in the distribution of rotavirus genotypes according to months.

When the distribution of rotavirus G and P genotype combinations according to age groups was analyzed, no G and P genotype combination was detected in 83.3% of foreign-born children under 2 years of age and 92.3% of children aged 2–5 years. There was no significant difference in genotype distributions between ages. There is no publication in the literature on genotype distribution according to age. These findings suggest that genotypes do not show a significant difference between gender and age groups.

### Conclusion

This study represents a unique and significant contribution to the literature as the first research to reveal the distribution of rotavirus genotypes among the foreign-born population living in the region in question. The findings obtained demonstrate that determining the circulating genotypes in rotavirus gastroenteritis plays a critical role, particularly in monitoring the antigenic structures targeted by vaccines and evaluating the effectiveness of vaccination programs. Indeed, the circulation of different genotypes can be decisive for the coverage and protective levels of existing vaccines.

However, rotavirus genotypes must be considered not only in terms of their molecular and epidemiological characteristics but also in terms of their clinical implications. Elucidating the relationship between genotype diversity and clinical parameters such as the clinical course of the disease, severity of symptoms, risk of complications, and need for hospitalization will provide important information for patient management and identification of risk groups. In this context, it is recommended that the possible relationships between genotype distribution and clinical findings be evaluated through multicenter and prospective studies.

Furthermore, the dynamic nature of rotavirus genetic structure and the potential for changes in genotype distribution over time highlight the necessity for long-term surveillance studies. Factors such as the expansion of vaccination programs, population mobility, and viral reassortment mechanisms are key drivers that can influence regional and global genotype composition. Therefore, conducting genotype surveillance studies covering different years and updated at regular intervals is of great importance for both monitoring epidemiological trends and shaping future vaccination strategies.
